# Age-Cohort Differences in Longitudinal Associations Between the Rate of Internet Use and Memory Functioning

**DOI:** 10.1093/geroni/igaa057.1321

**Published:** 2020-12-16

**Authors:** Vineet Raichur, Lindsay Ryan, Richard Gonzalez, Jacqui Smith

**Affiliations:** University of Michigan, Ann Arbor, Michigan, United States

## Abstract

Cross-sectional analyses of internet use patterns among older adults find that the rate of internet use is less with greater physical and memory difficulties. It is not clear, however, how age-cohorts differ in their internet use as physical and memory difficulties increase over time. In addition to factors such as increasing accessibility (cost) and social influences, the expansion and cognitive complexity of functions performed by the internet-enabled devices over time could influence internet use patterns. In this study, we investigate how the association between internet use and episodic memory difficulties over time varies between cohorts. We analyzed longitudinal data from the Health and Retirement Study (N = 15,703 in 2002; Aged 51 and older) between years 2002-2016 using mixed effects logistic regression models. Immediate and delayed word recall measures were used to assess episodic memory. Rate of internet use in the sample increased from 30% in 2002 to 53% in 2016. Rate of internet use among younger age groups was significantly higher in the baseline year. Younger age groups also showed a significantly higher rate of increase in internet use over time. In general, internet use decreased with episodic memory impairment. In addition to these effects, the effect of episodic memory on the rate of increase in internet use over time is lower in younger cohorts. These results indicate that younger cohorts of older adults are more likely to maintain internet use as they continue to age and therefore could better utilize technology for communication, social interactions and health interventions.

